# Influence of Firing Temperature on Phase Composition and Color Properties of Ceramic Tile Bodies

**DOI:** 10.3390/ma14216380

**Published:** 2021-10-25

**Authors:** Kornelia Wiśniewska, Waldemar Pichór, Ewelina Kłosek-Wawrzyn

**Affiliations:** Department of Building Materials Technology, Faculty of Materials Science and Ceramics, AGH University of Science and Technology, al. Mickiewicza 30, 30-059 Kraków, Poland; pichor@agh.edu.pl (W.P.); eklosek@agh.edu.pl (E.K.-W.)

**Keywords:** color, dolomite, cream-firing clays, temperature, phase composition

## Abstract

This study is focused on the behavior of the cream-firing clays from Opoczno region (Poland). The ceramic masses on which tests were carried out consisted of cream-firingBorkowice clay and dolomite in two different grain sizes as an additive that changes the color of ceramic materials. Test samples were prepared by plastic method and fired at range of 1100–1240 °C. Phase composition of theinvestigated materials was characterized by XRD method with quantitive analysis of the amorphous phase determined by the Retvield technique. Color properties of the surface of the obtained ceramic materials were determined in CIE-Lab color space. The phase composition of the obtained ceramics depends on the firing temperature. The color of the surface of the ceramic materials also depends on the firing temperature. There was a tendency to decrease the brightness, decrease the blue shade, and increase the yellow shade of the surface of materials with increasing the temperature. The conducted tests allowed to conclude that the color of ceramic materials depends on their phase composition. The most important role in the formation of color correspond to the amorphous phase, formed during the process. The lower content of the amorphous phase in the material allows to obtain brighter products with a lower proportion of yellow, and therefore closer to white. Moreover, following tests were carried out: total water absorption, total open porosity, linear shrinkage, and flexural strength. With increasing the temperature, total water absorption and total open porosity decrease, and total linear shrinkage increases due to the progressive sintering process. Flexural strength increases with the increase of the firing temperature for materials consisting of Borkowice clay. The addition of dolomite introduced new pores into the material, which resulted in an increase in flexural strength at lower firing temperatures and a decrease in flexural strength at higher firing temperatures.

## 1. Introduction

The exposure of phyllosilicates and other accompanying minerals such as quartz, feldspar, hematite, dolomite, and calcite to high temperature causes a series of phase transformations that affect the properties of the final ceramic material [1]. The formation of new crystalline phases is complex. During the phase transformation process, when the crystalline structures of minerals exceed their stability limits, they are partially decomposed, while the new crystalline structures are formed. The destruction of the pre-existing structure does not occur instantaneously [1]. During the firing process, the minerals contained in clays are structurally and chemically modified that significantly transformthe original clay materials. These transformations are due to the high temperature and low pressure which characterize the firing process and are dependent on the chemical and mineralogical compositions of the original clay, its grain-size distribution, the maximum heating temperature, heating rate, duration of firing and kiln atmosphere [2,3]. Phase transformations are also influenced by the chemical and mineralogical composition of the used additive and by its amount in the ceramic mass. The firing process of ceramic masses was widely researched and described in the literature. As described above, during firing, thermal decomposition of the majority of the original crystalline phases occurs (excluding quartz) while new crystalline phases (especially mullite) and chemically differentiated glassy phase are formed [4]. Transitional mullite is formed at 1075 °C, while above 1100 °C mullite is formed [5]. Glassy phase appears at 700 °C, but its intense formation begins at a temperature of 850 °C to 900 °C. Gehlenite and anorthite phases are formed during the firing of the ceramic masses, which contain illite, kaolinite, quartz, and calcite [6].

There are a number of studies that determine the effect of the firing temperature on the properties of ceramic materials. The influence of the firing temperature on the flexural strength of ceramic materials is known. Tests performed on ceramic masses consisting of illitic clays from Castellon (Spain: Guadalquivir valley) [7] and on ceramic masses consisting of Triassic clays collected from deposits in southern Tunisia [8] shows that the flexural strength of ceramic materials increases with increasing temperature. The above-mentioned studies [7,8] also proved that the higher the firing temperature of ceramic materials, the lower the porosity and water absorption of these materials. Researchers [9] determined the effect of the firing temperature on the frost resistance of ceramics. Authors proved that the frost resistance of ceramic materials increases with the increase in mechanical strength, which is related to the porosity of the materials. The lower the porosity of the materials, the more frost-resistant the materials are. Therefore, the frost resistance of ceramic materials increases with increasing temperature.

Influence of the dolomite addition to clays with a cream color after firing on the properties of the final ceramic products was already investigated [10]. The addition of dolomite applied to the ceramic mass made of cream-firing clay caused a change in the properties of ceramic materials. The grain size of the dolomite additive used has the main influence on the properties of ceramic materials. Moreover, ceramics with the addition of fine-grained or coarse-grained dolomite had different properties depending on the firing temperature. As the result of the experiment, an increase in water absorption and open porosity was observed for materials with the addition of a fine variety of dolomite flour, and a decrease in the value of these properties for materials with the addition of a coarse variety of dolomite flour (for materials fired at 1130 °C). The materials fired at 1150 °C are characterized by a decrease in the value of these properties, regardless of the type of dolomite used. In addition, a decrease in the mechanical strength of the ceramics with the addition of dolomite fired at 1130 °C and an increase in the mechanical strength of the ceramic materials with the addition of dolomite fired at 1150 °C was observed [10]. For comparison, dolomite addition also influences different clay types. For example, there is proof that the addition of dolomite in amount of 10% to the masses consisting of red-firing Triassic clay causes a significant improvement in the frost resistance of the final product and increases its mechanical strength [11].

Dolomite is used as an additive to ceramic masses due to the high content of CaO and MgO, which are fluxes. As a consequence, the addition of dolomite intensifies the sintering process, which leads to a change in the physicochemical properties of the materials. The benefits of using dolomite as an addition to ceramic masses include an increase in frost resistance [11] and mechanical strength [10,11] as well as a decrease in water absorption [10]. Therefore, the addition of dolomite extends the life cycle of bricks and ceramic tiles fired at an appropriate temperature. Thanks to the possibility of obtaining better frost resistance, mechanical strength, and water absorption properties, products with the addition of dolomite are suitable as façade masonry elements-exposed to external factors.

The aim of this study was to investigate the influence of the firing temperature on phase composition and color properties ceramic materials made of masses containing cream-firing clay and dolomite.

## 2. Materials and Methods

To prepare specimens fired at different temperatures, three types of ceramic mass were prepared. The composition of the masses is presented in Table 1.

Process of the preparation of the experimental samples from presented masses is shown in Table 2.

Two types of raw materials were used to prepare the ceramic masses. The main component of ceramic masses was lower Jurassic kaolinite-illite clay from Opoczno region in Poland (Borkowice clay). Borkowice clay has a kaolinite character and a creamy color after firing, as well as a low content of coloring oxides (0.99% Fe_2_O_3_ and 1.34% TiO_2_) [12]. The conclusion of the research was the determination that the favorable mineral and chemical composition of Borkowice clay determines the possibility of its application in the production of ceramic tiles. Borkowice clay can also be used as a supplement to the dificit base of white- and cream-firing clays. Ołdrzychowice dolomite from Lower Silesia region (Poland) in two different grain sizes was used as an addition to two ceramic masses.

Dolomite Ołdrzychowice is described as one of the purest varieties of dolomites from the Precambrian [13]. Two different grain sizes of dolomite samples were prepared by dry milling. The paper presents an analysis of the properties of raw materials performed as part of previous research [10]. Extensive raw materials analysis was carried out. Borkowice clay and Ołdrzychowice dolomite was subjected to chemical composition analysis by the XRF method (WDXRF AxiosmAX spectrometer, PANalytical, Malvern Panalytical Almelo, The Netherlands). Moreover, phase composition of raw materials was determined by the XRD method (X’Pert Pro, Phillips, Malvern Panalytical, Almelo, The Netherlands); with the following equipment parameters: radiation source: copper anode; angle range (2θ): 3–70°; angle step: 0.008°; scanning velocity: 193.04 s. Particle size distribution of Borkowice clay was determined byhydrometer analysis for soils and clays (in accordance with ASTM D7928–17: Standard Test Method for Particle Size Distribution (Gradation) of Fine-Grained Soils Using the Sedimentation (Hydrometer) Analysis); particle size distribution of Ołdrzychowice dolomite was characterized by laser diffraction analysis (Malvern Mastersizer2000 analyzer, Malvern Panalytical, Almelo, The Netherlands). Moreover, a thermal analysis of raw materials was carried out on the Netzsch Jupiter 449F3 derivatograph (Selb, Germany). Derivatograph enabled the simultaneous performance of differential thermal analysis (DTA), thermogravimetry (TG) and evolved gas analysis, which is necessary for the correct interpretation of the results. The measurement was carried out with the following parameters—heating range: 20–1100 °C; atmosphere: air (flow rate: 40 mL/min); sample size: about 100 mg; temperature rise rate: 100 °C/min; crucible material: corundum. Analysis of the results was carried out using Proteus Netzsch Thermal Analysis (Netzsch, Selb, Germany). The accuracy of the thermobalance used was: ±0.001 mg. The accuracy of the temperature reading was: ±0.01 °C. Determination of the properties of the final ceramic materials was the important aim of this project. Color of the surface of ceramic materials was determined using Colorimeter 3Color CP-10 (3Color^®^, JodłowaPoland) using CIELab color space with a CIE 10°/D65 observer. CIELab color space is characterized by three indicators: L–luminance (brightness), a-color from green to red, b-color from blue to yellow. The L*a*b color space was determined [14] as a 3D coordinate system. Luminescence values extend from 0 to 100, while the a and b axis scales are determined by the range of values from −150 to +100 and from −100 to +150, respectively. Shades of green and blue are assigned as negative numerical values (−), shades of red and yellow positive (+) [14]. Additionally, deltaE for obtained ceramic materials was also determined using a Colorimeter 3Color CP-10. Obtained materials properties, such as water absorption, total open porosity, total shrinkage, and flexural strength were also determined in accordance with European standards (PN-EN 14411:2016 Ceramic tiles—definition, classification, characteristics, assessment, and verification of constancy of performance and marking). The phase compositions of the finalmaterials were determined by XRD analyses (according to the method used for raw materials testing) with the quantitative analysis of the amorphous phase by the Rietveld method [15], using 10% of reference material (zincite). Additionally, the paper shows the microstructure of selected materials. The microstructure analyses were carried out using the SEM electron microscope Nano Nova SEM (FEI, Hillsboro, OR, USA) and presented in the images with ×500 magnification.

## 3. Results

### 3.1. Raw Materials Analysis

#### 3.1.1. Chemical Composition Analysis (XRF) of Raw Materials

Chemical composition of the raw materials (Borkowice clay and Ołdrzychowice dolomite) is shown in Table 3.

Chemical analysis of Borkowice clay shows a significant amount of Al_2_O_3_ (30.92%) and low content of coloring oxides: Fe_2_O_3_ and TiO_2_ (0.99% and 1.34%, respectively). XRF analysis of Ołdrzychowice dolomite shows large quantities of CaO (36.06%) and MgO (16.79%) and lesser amounts of coloring oxides: Fe_2_O_3_ and TiO_2_ (0.19% and 0.02%, respectively) than Borkowice clay [10].

#### 3.1.2. Phase Composition Analysis (XRD) of Raw Materials

**Figure 1:**
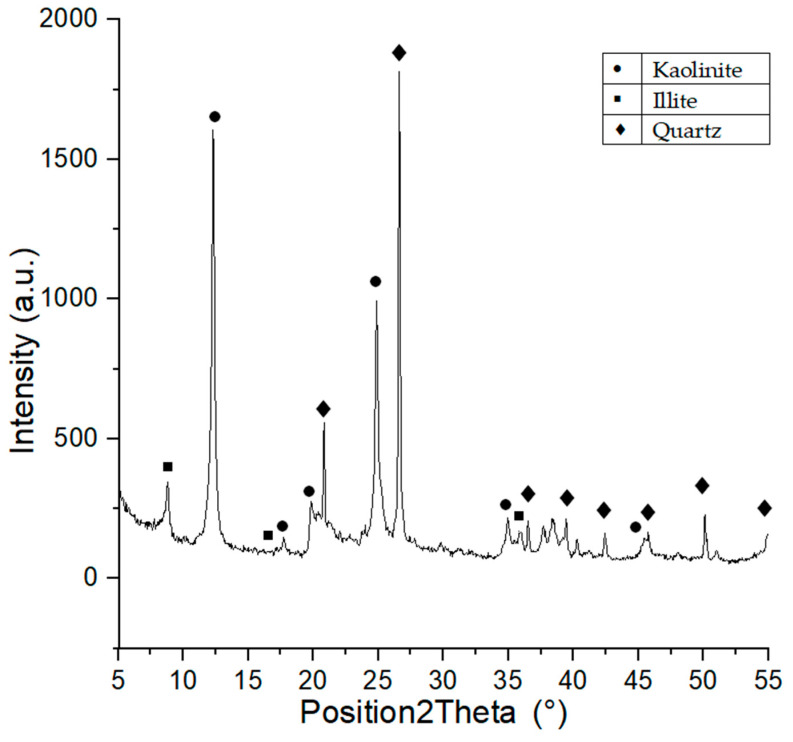
XRD diagram of Borkowice clay.

Phase composition of Borkowice clay indicates the presence of large quantities of kaolinite, illite, and quartz [10], which is a proof of the kaolinite-illite character of the raw material. The semiquantitive ratio of kaolinite:illite:quartz was calculated based on the XRD analysis using X’PertHighscore program. The result of this analysis shown that semiquantitivekaolinite:illite:quartz ratio was 5:4:1. Moreover XRD analysis is shown in Figure 1. Ołdrzychowice dolomite is characterized by a significant content of dolomite and lesser contents of calcite and quartz. It was calculated that the raw material is characterized by 95% dolomite content [10], which proves that the Ołdrzychowice dolomite is considered one of the purest varieties of dolomite [13].

#### 3.1.3. Particle Size Distribution of Raw Materials

**Figure 2:**
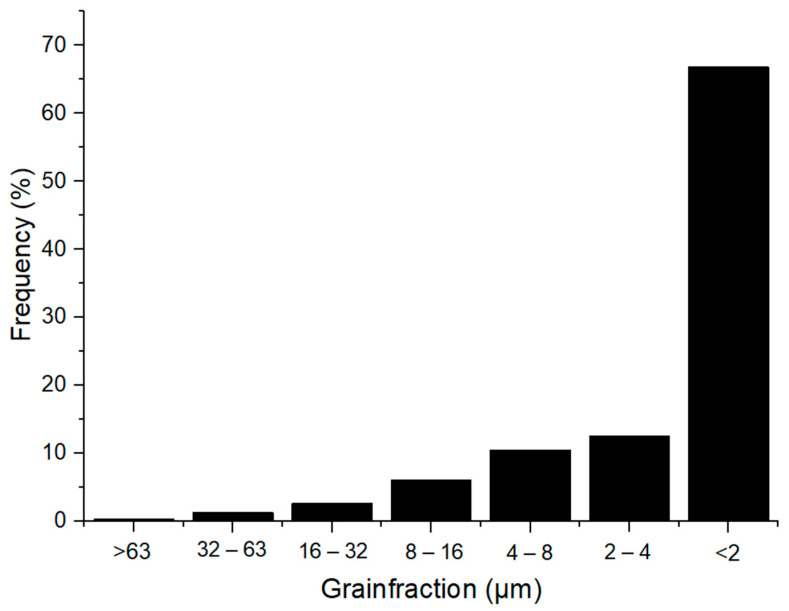
Granulometric curve of Borkowice clay [10].

**Figure 3:**
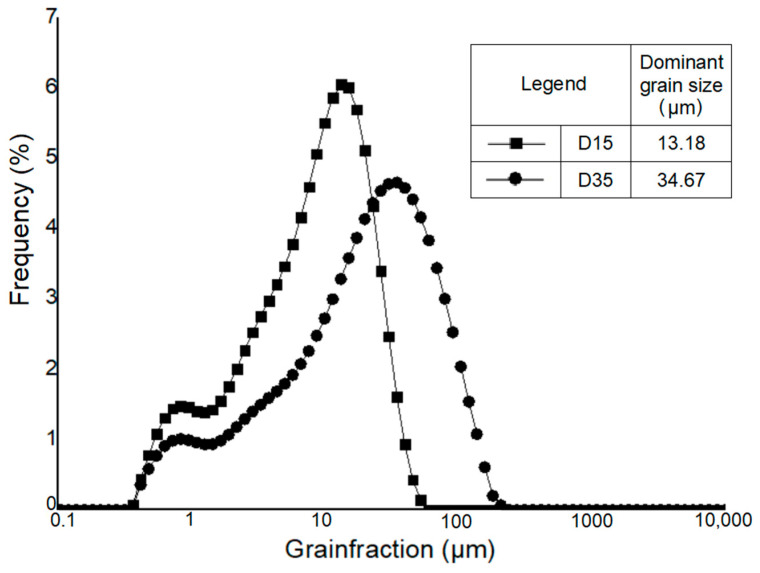
Granulometric curve of Ołdrzychowice dolomite in different granulation [10].

The particle size distribution of Borkowice clay and Ołdrzychowice dolomite is shown in Figure 2 and Figure 3.

Hydrometer analysis of Borkowice clay determined that the largest share of grains is characterized by a size smaller than 2 μm due to a high content of clay minerals (especially kaolinite). In contrast, in the grain size range of 2 to 63 μm, dolomite flours used as an additive are characterized by a decrease in the share of grains as their size decreases. Ołdrzychowice dolomite flours were used, as an additive to ceramic masses in two different granulations. The raw material D15 is characterized by the dominant grain size of 13.18 μm, whereas the raw material D35 is characterized by the dominant grain size of 34.67 μm.

#### 3.1.4. Thermal Analysis of Raw Materials

**Figure 4:**
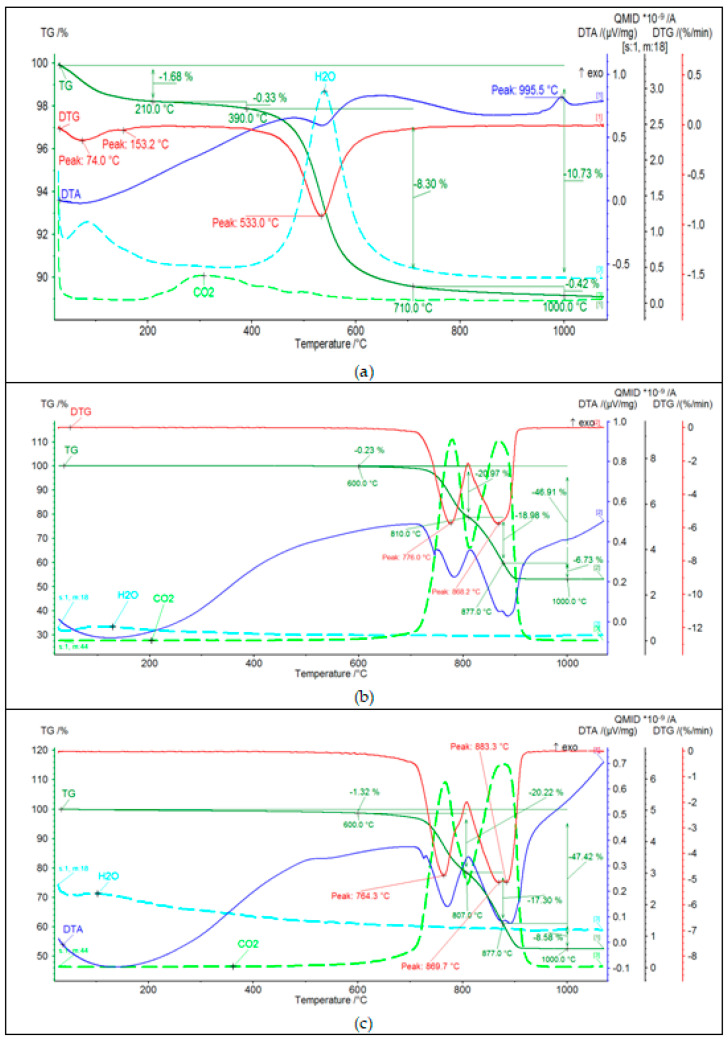
Thermal analysis (DTA, TG, DTG, and EGA patterns) of (**a**) Borkowice clay, (**b**) Ołdrzychowice dolomite: 13.18 μm, (**c**) Ołdrzychowice dolomite: 34.67 μm.

Results of thermal analysis of Borkowice clay and Ołdrzychowice dolomite in two different grain sizes is shown in Figure 4.

Based on the analysis of the DTA curve of the Borkowice clay (Figure 4a), it was found that the raw material contains mainly kaolinite, as two thermal effects characteristic for this mineral were recorded. The first thermal effect is the endothermic effect at about 550 °C, related to the dehydroxylation process of kaolinite and illite, which is also confirmed by the analysis of the EGA curve. The second effect is an exothermic effect at 995 °C, resulting from the synthesis of a new spinel-like phase, due to the kaolinite reaction. It was observed on the TG curve that in the temperature range from 210 °C to 1000 °C there was a multiple weight loss. The greatest weight loss occurred in the temperature range from 390 °C to 710 °C (8.30%), due to the dehydroxylation of clay minerals. An endothermic effect at 153 °C was observed in the DTA curve dueto the dehydration of illite. Therefore, it can be concluded that the clay minerals present in the tested raw material are kaolinite and illite. Moreover, based on the analysis of the EGA curve, a small amount of organic matter (0.33%) was found. This is evidenced by the release of CO_2_ in the temperature range from 210–390 °C. Based on the analysis of DTA curves of Ołdrzychowice dolomite in two differend grain sizes (Figure 4b,c), the main mineral component of this raw material is dolomite, as two endothermic effects characteristic for this mineral were recorded: at about 760 °C and about 880 °C. These effects are due to the two-stage thermal dissociation of the carbonate. In the first stage, CaCO_3_, MgO, and CO_2_ are formed in the temperature range from 700–750 °C. In the second stage, the following oxides are formed: MgO and CO_2_ [16]. The first endothermic effect was shifted towards higher temperatures for the raw material with a coarser grain size. The first endothermic effect illustrates the decomposition of calcium carbonate, which is an admixture in the raw material or is formed by recombination of CaO and CO_2_ [17]. Dehydrocylation, mineral transformation and glass formation are thermal processes that are proceeding at temperatures characteristic for raw materials. Different parameters can affect thermal reactions during the firing process, such as nature and crystalline structure of raw materials and the conditions of the sample analysis process, as well as sample weight and the particle size distribution of the material [18].

### 3.2. Material Properties Analysis

**Figure 5:**
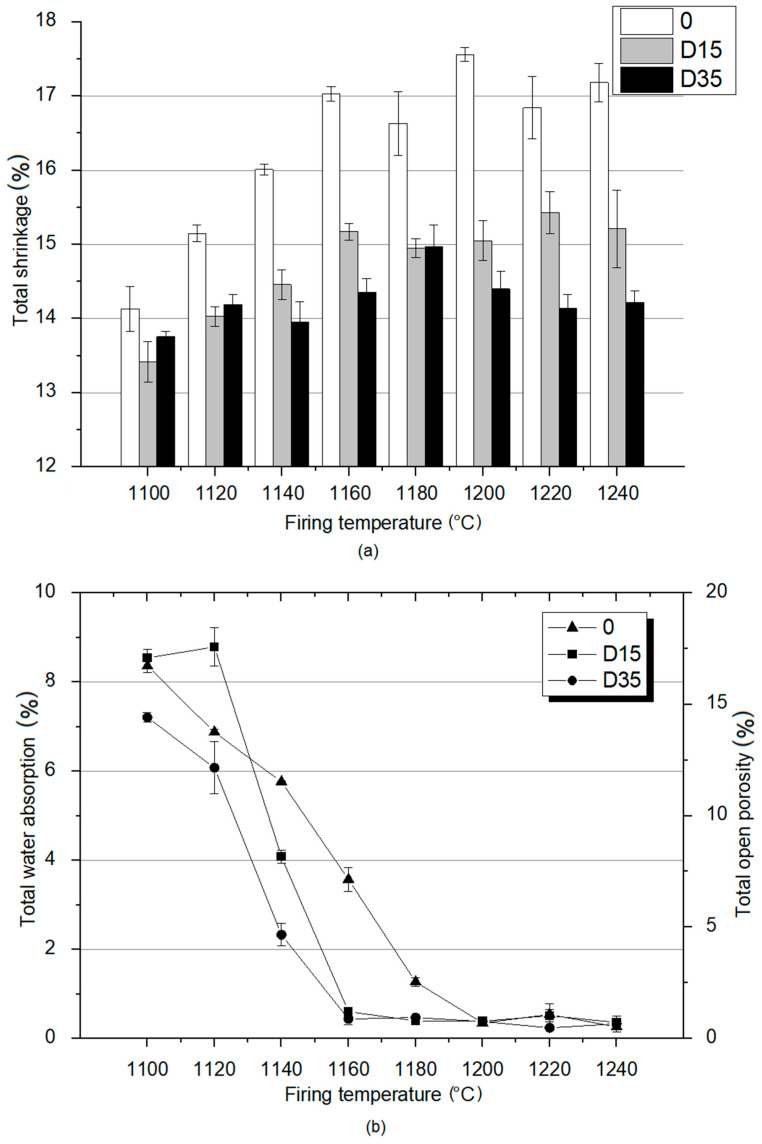
Material properties analysis of obtained ceramic materials: (**a**) total shrinkage; (**b**) total water absorption and total open porosity.

As part of the study of the material properties of the fired ceramics, total shrinkage was determined as well as total open porosity and total water absorption for these materials. The results of these analyzes are shown in Figure 5.

The analysis of the results presented in Figure 5a showed that the reference material 0 is characterized by the highest shrinkage at each temperature. The highest value of shrinkage for the reference material 0 was recorded at 1200 °C, and the lowest at the lowest temperature of 1100 °C. These results show that the reference material is characterized by a higher degree of sintering than plastics with the addition of dolomite (D15, D35), regardless of the firing temperature. The shrinkage for the reference material increases with the temperature increase from 1100 °C to 1160 ℃, and then its values remain at a similar level from 1160–1240 °C. D35 material is characterized by higher total shrinkage than D15 material at lower temperatures—1100 °C and 1120 °C. At higher temperatures, D35 is characterized by lower shrinkage values than D15. At the temperature of 1180 °C, the shrinkage of both materials with the addition of dolomite fluctuates around similar values. The presented analysis proves that the sintering process at lower temperatures is more intensive for the material with the addition of coarse-grained dolomite and at higher temperatures for the material with the addition of fine-grained dolomite. The total shrinkage value for the D15 material, similarly to the reference material, increased with the temperature increase from 1100 °C to 1160 °C, and then remained at a similar value. Total shrinkage values for D35 material increase from 1100 °C to 1180 °C, and then decrease from 1180 °C to 1240 °C. Studies showed that for kaolin clays, shrinkage increases linearly with increasing temperature due to the formation of the vitreous phase [19]. On the other hand, another experiment showed that ceramic mixtures containing raw material additives with calcium oxide and magnesium oxide in their chemical composition can be resistant to shrinkage during the firing process. The probable cause of this phenomenon is that in thick-crystalline fused clinkers, low-melting calcium compounds are found in small clusters between large crystals of calcium oxide and magnesium oxide, which makes the entire structure stiff and resistant to shrinkage during the firing process [20]. The conclusions presented above correspond to the results obtained during the research. For the reference material, the shrinkage increases linearly with the temperature increase, and for the materials with the addition of dolomite, no linear changes were observed.

Total water absorption and related total open porosity are important indicators for determining the properties of sintered materials. Based on these parameters, it is possible to determine the suitability of ceramic tiles for outdoor or indoor use. The results of total water absorption and total open porosity tests are shown in Figure 5b. Based on the data analysis, at 1100 °C and 1120 °C, the total water absorption values for all materials (for the reference material and for materials with dolomite addition) oscillate around similar values. At 1140 °C, reference material is characterized by the highest value of total water absorption. D15 material is characterized by lower value of the total water absorption. Furthermore, for D35 material, a much lower water absorption value was recorded compared to that of other materials. At higher temperatures, D15 and D35 materials are characterized by much lower water absorption values compared to that of the reference material. This means that the addition of dolomite reduces water absorption and thus reduces the open porosity. Additionally, the use of coarse-grained dolomite as an additive reduces total water absorption at a lower temperature (1140 °C) than the use of fine-grained dolomite (1160 °C). Moreover, the reference material is characterized by a level of total water absorption similar to materials with the addition of dolomite only at a temperature of 1200 °C. The analysis of the data contained in Figure 5b shows that the materials with the addition of dolomite achieve the minimum open porosity at 1160 °C and the reference material at 1200 °C. For this reason, the addition of dolomite has a positive effect on the properties of the fired products, as the low value of water absorption allows the use of clinker tiles outside of buildings.

**Figure 6:**
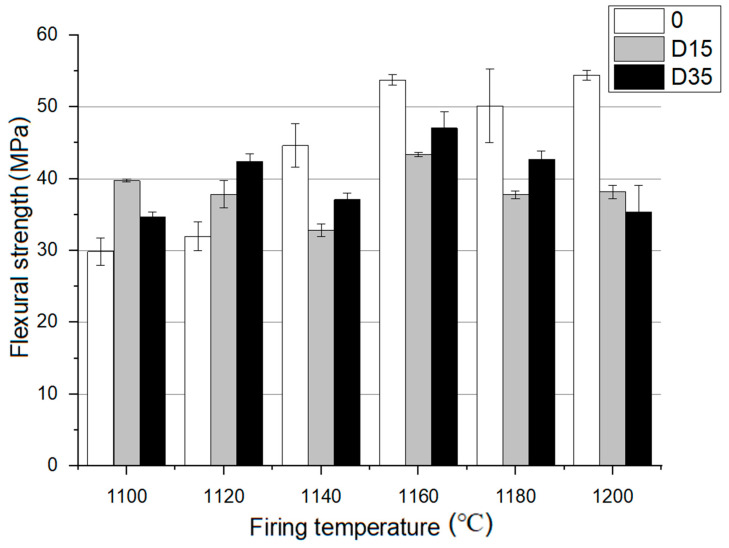
Flexural strength analysis for materials fired at 1100 °C to 1200 °C.

Additionally, flexural strength of the materials fired at 1100 °C to 1200 °C was tested. The results of this analysis are presented in Figure 6.

At 1100 °C, D15 material is characterized by the highest flexural strength, and at 1120 °C, D35 material is similarly characterized. At higher temperatures: 1140–1200 °C the reference material is characterized by the highest flexural strength. For the reference material, flexural strength increases with the increase of the firing temperature. D15 material is characterized by a decrease in flexural strength from 1100–1140 °C. At 1160 °C, flexural strength increases for D15 material, and then it decreases from 1160–1200 °C. For D35 material, no effect of temperature on flexural strength was noticed. Ceramics with the addition of dolomite are characterized by the highest value of bending strength at a temperature of 1160 °C. The reference material is characterized by the highest value of flexural strength at the highest temperature, 1200 °C. Based on the data analysis, the addition of dolomite at lower temperatures of 1100 °C, 1120 °C causes an increase in flexural strength, while at higher temperatures 1140 °C, 1160 °C, 1180 °C, 1200 °C, there is a decrease in flexural strength compared to that of the reference material.

### 3.3. Material Properties Analysis

**Figure 7:**
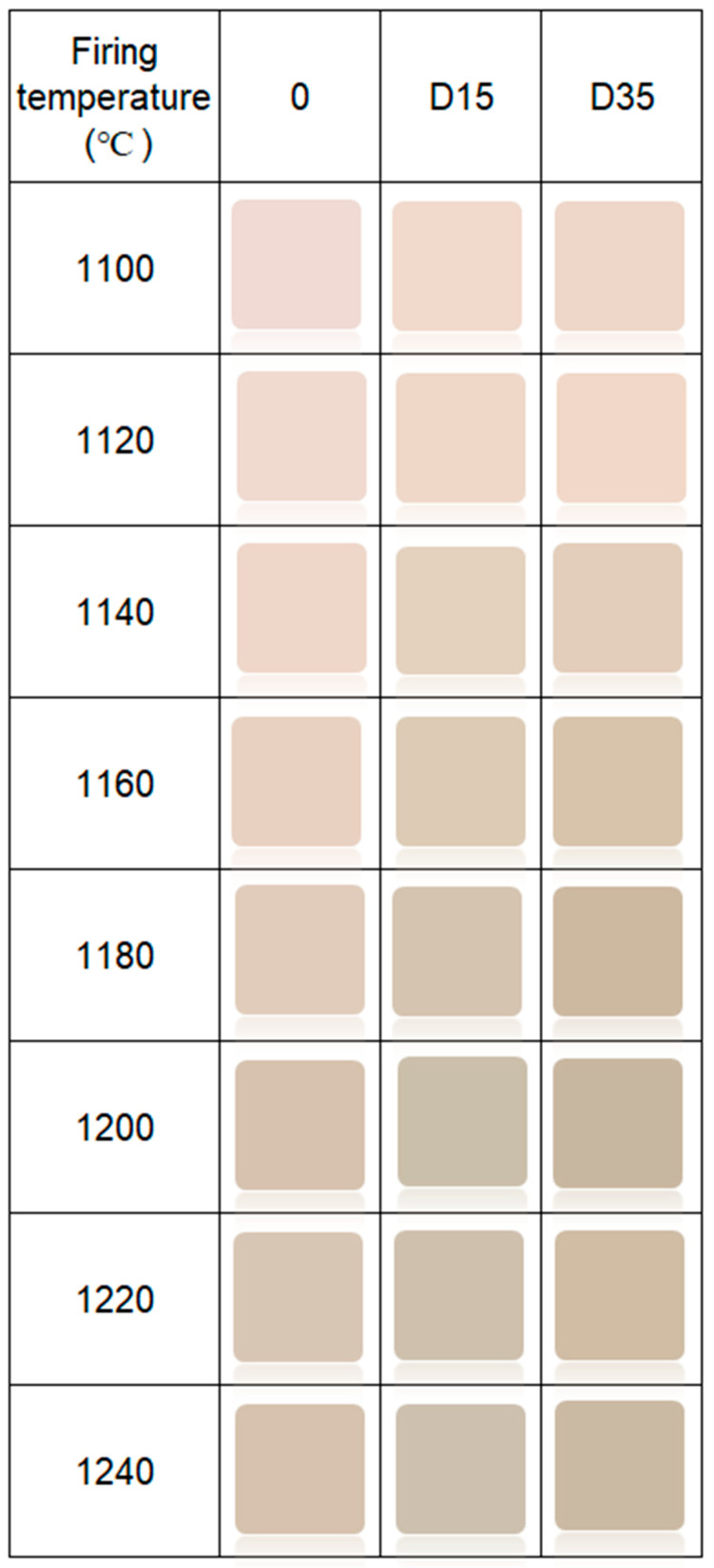
Color of surface of obtained ceramic materials.

Color of the surface of ceramic materials is presented in Figure 7.

**Figure 8:**
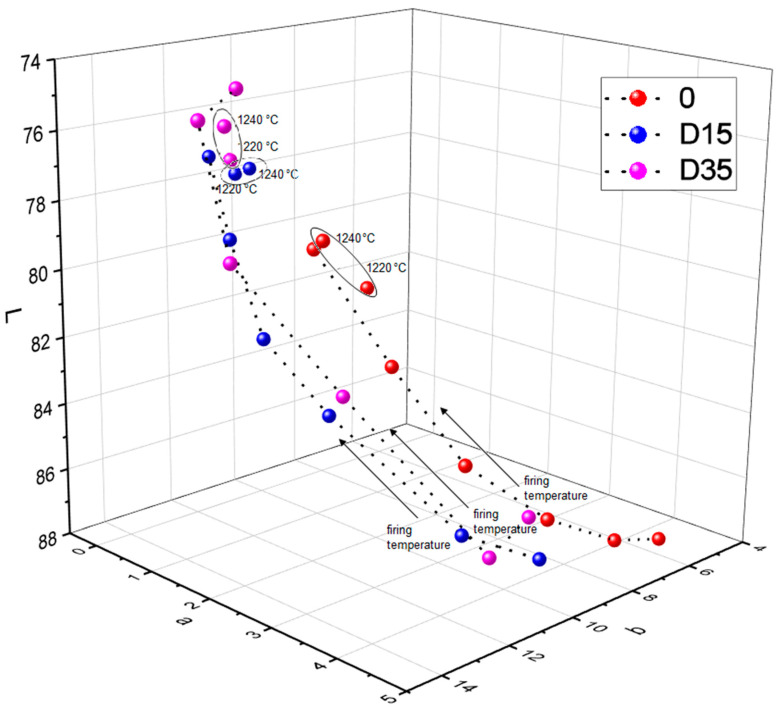
Dependence between L, a, b color indicators and firing temperature of ceramic materials.

The addition of dolomite causes a color change of ceramic materials. Moreover, the firing temperature also influences the color of the surface of obtained ceramic materials. Figure 8 shows the dependence between the L*a*b color indicators and the firing temperature of ceramic materials.

Based on Figure 8, the dependence between the L indicator and the firing temperature, the following conclusion was found: from 1100 °C to 1200 °C the value of the L indicator decreases for all tested materials. Then, between 1200 °C and 1220 °C, the L indicator increases”. Between the temperature of 1220 °C and 1240 °C there is another decrease of the L indicator for materials 0 and D35 and the stabilization of the L indicator for material D15. The analysis of dependence of the value of the a indicator on the firing temperature of ceramic materials showed the following relationship: with increasing the firing temperature (from 1100 °C to 1200 °C), the value of the a indicator decreased, and then, from 1200 °C to 1240 °C, the value of the a indicator stabilized for materials 0 and D15. For D35 material, the a indicator stabilized at lower temperature of 1180 °C. Various relationships were observed for the reference material 0 and for materials with the addition of dolomite during the analysis of dependence of the value of the b indicator on the firing temperature of ceramic materials. The value of the b indicator for the reference material 0 increased with increasing the firing temperature, except for the temperature of 1220 °C, where the value of the b indicator decreased. The value of b indicator for the material with addition of finer variety of dolomite–D15 increased with increasing the firing temperature, from 1100 °C to 1160 °C, and then decreased with increasing the firing temperature, from 1160 °C to 1240 °C. The value of b indicator for the material with addition of coarser variety of dolomiteD35 increased with increasing the firing temperature, from 1100 °C to 1180 °C, then decreased and stabilized at the temperature range from 1200 °C to 1240 °C. To sum up, in the firing temperature range from 1100 °C to 1200 °C, the following dependences occur: as the firing temperature increases, the value of the b indicator increases, and the values of a and L indicators decrease. For materials fired at 1220 °C and 1240 °C, these dependences lose their linear character. To fully determine the color change of ceramic materials with a change in firing temperature, deltaE was determined between the same types of materials fired at adjacent temperatures. The results are presented in Table 4.

The analysis of the data contained in Table 4 shows that the color change between the temperatures of 1100 °C and 1120 °C is similar for all types of ceramic materials. For the reference material 0, the color change between individual, adjacent temperatures is on a similar level. The greatest color change for this material was recorded between the temperatures of 1180 °C and 1200 °C. Materials with the addition of dolomite are characterized by the greatest color change between the temperatures of 1120 °C and 1140 °C. Color change for material with the addition of finer dolomite at higher temperatures gradually decreases. There is an insignificant color change from 1200 °C to 1240 °C. However, for the material with the addition of coarser dolomite, color change is significant also between the temperatures 1140 °C and 1160 °C, and 1160 °C and 1180 °C. The color change in this material is insignificant for the temperature range from 1220 °C to 1240 °C.

### 3.4. Phase Composition Analysis

The analysis of the phase composition with the determination of the amount of amorphous phase was carried out for materials 0, D15, and D35 fired at the following temperatures: 1140, 1200, and 1240 °C. These materials were selected on the basis of the analysis of the color results and the characteristic changes in the L*a*b color indicators that occurred at the following temperatures: 1140, 1200, and 1240 °C (Figure 8). The results of the phase composition analysis for the above-mentioned materials are presented in Table 5.

**Figure 9:**
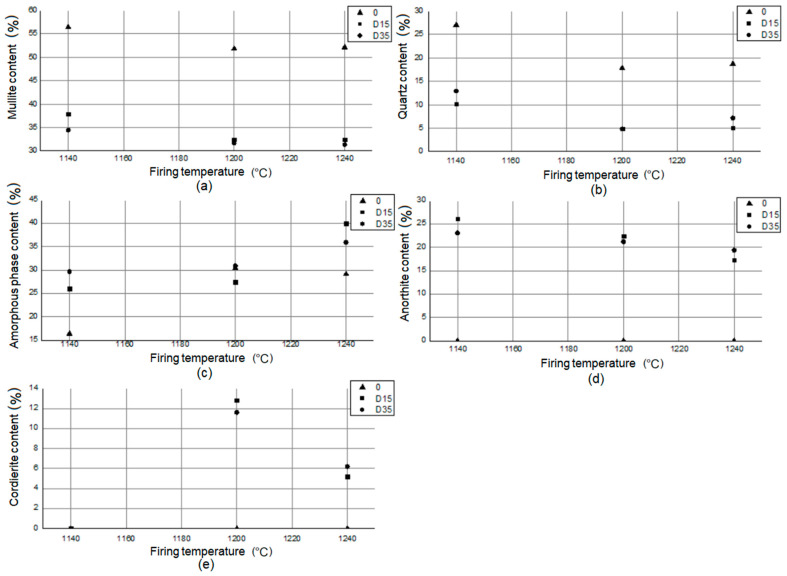
Relationship between (**a**) mullite, (**b**) quartz, (**c**) amorphous phase, (**d**) anorthite, and (**e**) cordierite content in phase composition of materials and firing temperature.

On the basis of the data contained in the table, the amount of mullite (Al_4+2x_Si_2−2x_O_10−x_) in the reference material 0 is higher than in the materials with the addition of dolomite: D15, D35, regardless of the firing temperature. This proves a higher sintering level of the reference material compared to materials with the addition of dolomite. The same relationship exists for quartz (SiO_2_). In the reference material, the amount of this phase is much greater than in materials with the addition of dolomite. In plastics with the addition of dolomite: D15, D35, there is a significant amount of anorthite (Ca[Al_2_Si_2_O_8_]). There is no anorthite in the reference material. Anorthite in materials with the addition of dolomite appears as a result of reactions involving calcium oxide, which is present in the chemical composition of dolomite (Table 3). At higher temperatures (1200 °C, 1240 °C), another new phase appears in materials D15, D35: cordierite (Mg_2_Al_3_[AlSi_5_O_18_]). Cordierite in materials with addition of dolomite appears as a result of reactions involving magnesium oxide, which is present in the chemical composition of dolomite (Table 3). Magnesium is present in the tested materials as a component of amorphous phase and as separate crystalline phase: cordierite. Stoichiometric cordierite is synthesized at temperatures higher than 1430 °C [21]. However, there are studies that show the possibility to prepare cordierite ceramics by ball milling and reaction sintering of kaolinite and synthetic magnesia in lower temperature [22]. Experiments proved that different crystalline phases are presented in materials sintered at 900–1150 °C and cordierite started to crystallize at 1200 °C [22]. According to Table 5 cordierite appeared in the phase composition of materials with the addition of dolomite also at 1200 °C. Based on the literature data [23], cordierite is the main phase formed during the crystallization of glasses in the MgO-Al_2_O_3_-SiO_2_ system. The presence of TiO_2_ in the chemical composition (Table 3) drives the cordierite crystallization process [23]. The presence of magnesium in the vitreous and corundum phases improves the mechanical properties of the material [24], its mechanical strength and frost resistance [11]. There is no relationship between the type of material and the amount of amorphous phase present in its phase composition. The smallest amount of amorphous phase was observed for the material 0 fired at 1140 °C (16.4%), and the highest for the D15 material fired at 1240 °C (40.0%). Figure 9 shows the relationship between the phase composition of the materials and the firing temperature.

The analysis of the dependency graphs allowed for a series of conclusions. As the temperature increases (1140–1200 °C), the amount of mullite decreases, and then it remains constant (1200–1240 °C) in the phase composition of the materials. As the temperature increases (1140–1200 °C), the amount of quartz decreases, and then (1200–1240 °C) it increases in the phase composition of the materials. For materials with the addition of dolomite (D15, D35), the amount of amorphous phase in the phase composition increases with increasing temperature (1140–1240 °C). Between 1140 °C and 1200 °C, the amount of amorphous phase in the phase composition increases slightly, and from 1200 °C to 1240 °C, it increases significantly. For the reference material 0, the amount of amorphous phase in the phase composition of the material increases significantly from 1140 °C to 1200 °C, and then it decreases slightly from 1200 °C to 1240 °C. Anorthite does not appear in the phase composition of the reference material 0. The amount of anorthite in the phase composition of materials with addition of dolomite (D15, D35) decreases with increasing temperature. Cordierite does not appear in the phase composition of the reference material 0. Cordierite does not appear in the phase composition of materials with the addition of dolomite (D15, D35) fired at 1140 °C. Cordierite appears in the phase composition of materials with the addition of dolomite (D15, D35), fired at 1200 °C and 1240 °C, and its amount reduces twice as the temperature increases.

#### 3.4.1. Dependence between Flexural Strength of Obtained Ceramic Materials and Mullite Content in Their Phase Composition

**Figure 10:**
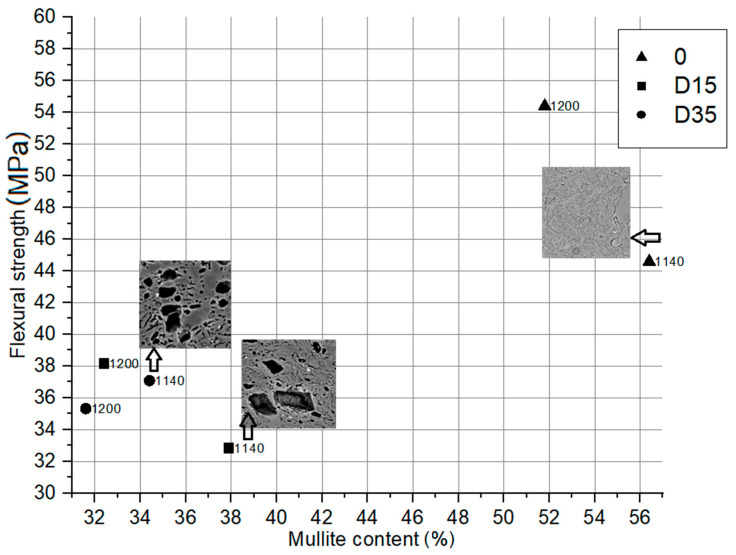
Dependence between flexural strength of obtained ceramic materials and mullite content in their phase composition.

The dependence between flexural strength of obtained ceramic materials and mullite content in their phase composition with microstructure analysis of selected materials is shown in Figure 10.

Based on the analysis of the diagram in Figure 10, it can be concluded that materials with a higher mullite content in their composition are characterized by a higher value of flexural strength. The reference material “0”, whose composition is characterized by a higher mullite content compared to the dolomite-containing materials, is characterized by the highest flexural strength. However, the presented relationship is not linear. On this basis, apart from the phase composition, there are also other factors that influence the flexural strength, such as microstructure of materials. The addition of dolomite additive to the ceramic mass influences its microstructure, as shown in Figure 9. During firing, the dolomite grains leave a large amount of closed pores, the size of which depends on the size of the dolomite grain introduced. In these pores there is unreacted dolomite residue, the presence of which determines the reduction of flexural strength. The flexural strength of materials with the addition of dolomite depends on the arrangement of pores with dolomite residue in the aluminosilicate matrix, the size of the pores and the dolomite conversion degree.

#### 3.4.2. Dependence between Color and Phase Composition of Obtained Ceramic Materials

**Figure 11:**
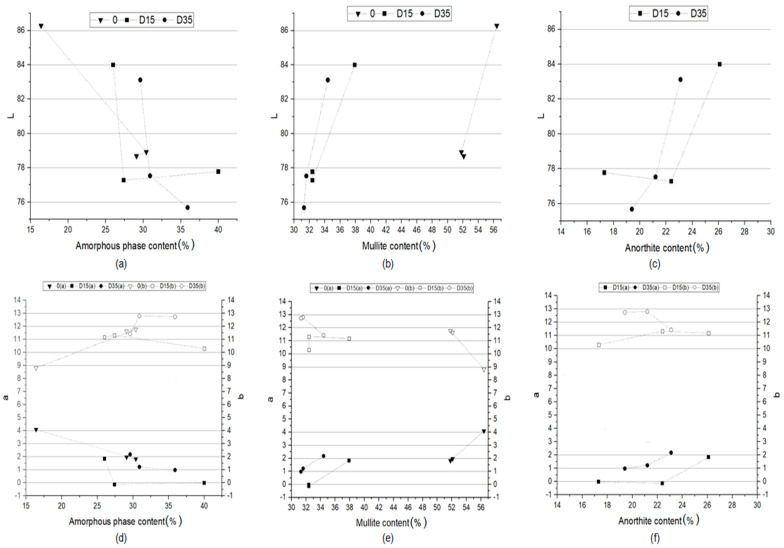
Dependence between *a* indicator and (**a**) amorphous phase content, (**b**) mullite content, (**c**) anorthite content and dependence between *b* indicator and (**d**) amorphous phase content, (**e**) mullite content, (**f**) anorthite content in the phase composition of obtained ceramic materials.

The color of the obtained ceramic materials is closely related to the phase composition of these materials. Figure 11 shows the dependence of the L*a*b color indicators of ceramic materials on the amount of amorphous phase, mullite, and anorthite in these materials.

The more amorphous phase in the phase composition of the materials, the lower the value of the L indicator, which is responsible for the lightness of the ceramic product (Figure 11a), and the lower the value of the a indicator (Figure 11d). This proves that the more amorphous phase in the phase composition of the material, the closer its color is to green and the material is darker. The increase in the proportion of the amorphous phase in the phase composition results in an increase in the b indicator, i.e., the formation of a yellow shade of color (Figure 11d). The lower content of the amorphous phase in the material allows for obtaining products with a lower proportion of yellow color, and therefore closer to white. The determination of the dependence of the L*a*b color indicators on the amount of mullite in the phase composition of the materials was used to determine the influence of the sintering process on the color of the materials. There is a similar relationship for all materials analyzed separately. The higher the content of mullite in the phase composition, the higher the value of the L indicator (lightness of the material) (Figure 11b) and the higher the value of the a indicator (the color is approaching red) (Figure 11e). The content of mullite in the phase composition has no significant influence on the value of the b indicator. Figure 11c,f show the dependence of the L*a*b color indicators on the amount of anorthite in the phase composition of the materials with the addition of dolomite. The higher the dolomite content, the higher the value of the L indicator, i.e., the lightness of the material (Figure 11c), as well as the higher the value of the a indicator (Figure 11f). No such relationship was observed for the material with the addition of dolomite with a finer grain (D15). Summing up, the phase composition of materials influences primarily the L and a color indicators, while the b indicator is influenced to a lesser extent. The most important role in the formation of color correspond to the amorphous phase, formed during the process.

## 4. Discussion

The research carried out as a part of experiment shows the influence of firing temperature on phase composition, and therefore, on color and technological properties of sintered ceramic materials based on Borkowice clay with addition of dolomite. The conducted research of the color properties of the surface of obtained ceramic materials allows to conclude that with the temperature increase from 1100 °C to 1200 °C, the L indicator andthe a indicator decrease, and the b indicator increases. This means that as the temperature increases, the surface of the ceramic materials becomes darker, and the proportion of green and yellow colors increases. These dependencies are strongly related to the phase composition of these materials. The performed experiment states that with the increase of the firing temperature, the amount of mullite in the phase composition of all analyzed materials decreases, and remains at a constant level above 1200 °C. Therefore, for materials fired at lower temperatures, a lower L indicator was recorded. The higher the firing temperature, the darker the surface of the material. Moreover, the amount of mullite in the phase composition determines the flexural strength of materials. However, this is not the only factor that affects the flexural strength. The second factor is the grain size of the dolomite additive. On the other hand, the amount of amorphous phase increases with increasing temperature. The increase in the amount of amorphous phase with increasing temperature is greater for materials with the addition of dolomite. The conducted research allowed to state that the amount of amorphous phase has a great influence on the color of the materials. As the amount of amorphous phase increases, the L indicator decreases-the surface of the material is darker. Moreover, the a indicator decreases and the b indicator increases. Therefore, with the increase in the amount of amorphous phase in the phase composition of the materials, i.e., with the increase of temperature, the lightness of the material decreases, and the share of green and yellow colors increases. With increasing temperature, the amount of anorthite in the phase composition of the ceramics with the addition of dolomite decreases. The less anorthite in the phase composition of the ceramic materials, the lower the value of the L indicator and the lower the value of the a indicator. The surface of the material becomes darker with increasing temperature, and its color becomes green. The presented analysis was confirmed by observing the test surface with the naked eye.

## 5. Conclusions

The addition of dolomite to the ceramic mass causes a change in the phase composition of fired sintered materials. Thus, the addition of dolomite changes the color of the material’s surface. This change is characterized by a different intensity, which depends on the firing temperature. Brightness, blue shade, and yellow shade of the color of the surfaces of the final materials were decreased with the increasing of the sintering temperature. The mechanism of color formation in connection with the phase composition of materials was proven. Formation of the color was mainly determined by the amorphous phase present in the phase composition. The lower content of the amorphous phase in the material allows to obtain brighter products with a lower proportion of yellow, and therefore closer to white. In addition, the change in the phase composition of materials with the addition of dolomite affects the technological properties of finished products—reduction of shrinkage, total water absorption and total open porosity, as well as increased strength-for materials fired at lower temperatures and reduced strength-for materials fired at higher temperatures. The research shows the role of the firing temperature, on which the formation of new crystalline phases and the characteristics of the amorphous phase depend on the shaping of the color of the surface of sintered ceramic materials.

## Figures and Tables

**Table 1:** Composition of ceramic masses.

Name of the Material	Amount of Raw Material (% by Mass)	
	BorkowiceClay	OłdrzychowiceDolomite
0	100	0
D15 *	90	10
D35 *	90	10

* Dolomite characterized by two different degrees of granulation.

**Table 2:** Preparation process of experimental samples.

**Method and Laboratory Equipment**	**Formation**		**Drying Process**	**Firing Process**	
	Vented laboratory screw press Plastic method		Dried in the room temperature (at about 20 °C) for 24 h–samples positioned at proper distance to allow air circulation Laboratory dryer	Laboratory electric kiln	
	**Samples characteristics:**				
	Beams with dimensions: 150 × 30 × 20 mm	Bricks with dimension: 75 × 30 × 20 mm			
**Conditions**	24 h storage of the masses in humid conditions		Dried to the constant mass	Oxidizing atmosphere	
**Process characteristics**	-		(I.) 35 °C for the first 6 h	Firing rate: 100 °C/h	
			(II.) Increase in the temperature to: 50 °C, 75 °C and 105 °C every 2 h	Dwelling time: − 1 h at selected temperatures: 100 °C, 600 °C, 900 °C − 2 h at maximum temperature	
			(III.) 105 °C for 7 h	**Maximum temperature (°C):**	
				T1	1100
				T2	1120
				T3	1140
				T4	1160
				T5	1180
				T6	1200
				T7	1220
				T8	1240

**Table 3:** Chemical composition of Borkowice clay and Ołdrzychowice dolomite [10].

Chemical Composition (wt/%)													
Raw Material	SiO_2_	Al_2_O_3_	Fe_2_O_3_	TiO_2_	CaO	MgO	K_2_O	Na_2_O	Cr_2_O_3_	P_2_O_5_	MnO	SO_3_	Loss of Ignition
Borkowice clay	53.22	30.92	0.99	1.34	0.23	0.41	1.60	0.16	0.02	0.15	0.00	0.02	10.73
Ołdrzychowice dolomite	1.39	0.36	0.19	0.02	36.06	16.79	0.06	0.06	0.00	0.01	0.06	0.01	44.95

**Table 4:** DeltaE between obtained ceramic materials fired at different temperatures.

Temperature Range (°C)	Name of the Ceramic Materials		
	0	D15	D35
	DeltaE between Ceramic Materials Fired at Different Temperatures		
1100–1120	1.4802	1.4995	1.4342
1120–1140	2.2342	3.4520	4.4325
1140–1160	2.2138	2.4182	4.6030
1160–1180	2.4945	2.6202	3.9185
1180–1200	3.8175	2.2117	1.3143
1200–1220	2.0315	0.6427	1.9094
1220–1240	2.1461	0.6733	0.9135

**Table 5:** Phase composition analysis for obtained ceramic materials fired at 1140, 1200, and 1240 °C.

Type of Material	0	D15	D35	0	D15	D35	0	D15	D35
Firing Temperature (°C)	1140			1200			1240		
Mullite (Al_4+2x_Si_2−2x_O_10−x_)	56.4	37.9	34.4	51.8	32.4	31.6	52.1	32.4	31.3
Quartz (SiO_2_)	27.0	10.1	12.9	17.8	4.9	4.8	18.7	5.0	7.1
Cordierite (Mg_2_Al_3_[AlSi_5_O_18_])	0.0	0.0	0.0	0.0	12.8	11.6	0.0	5.2	6.2
Anorthite (Ca[Al_2_Si_2_O_8_])	0.0	26.1	23.1	0.0	22.4	21.2	0.0	17.3	19.4
Amorphous phase	16.4	26.0	29.6	30.4	27.4	30.9	29.1	40.0	35.9

## Data Availability

The data is contained within the article. Additional data are available on request from the corresponding author.

## References

[1] Jordán M.M., Boix T. (1999). Firing transformations of cretaceous clays used in the manufacturing of ceramic tiles. Appl. Clay Sci..

[2] Maggetti M., Olin J.S. (1982). Phaseanalysis and its significance for technology and origin. Archaeological Ceramics.

[3] Moropoulou A., Bakolas A., Bisbikou K. (1995). Thermal analysis as a method of characterizing ancient ceramic technologies. Thermochim. Acta.

[4] Dondi M., Ercolani G., Melandri C., Mingazzini C., Marsigli M. (1999). The chemical composition of porcelain stoneware tiles and its influence on microstructural and mechanical properties. Intercam.

[5] Lecmote-Nana G.L., Bonnet J.P., Blanchart P. (2011). Investigation of the sintering mechanisms of kaolin-muscovite. Appl. Clay Sci..

[6] González-García F., Romero-Acosta V., García Ramos G., González Rodríguez M. (1990). Firing transformations of mixtures of clays containing illite, kaolinite and calcium carbonate used by ornamental tile industries. Appl. Clay Sci..

[7] Jordán M.M., Montero M.A., Maseguer S., Sanfeliu T. (2008). Influence of firing temperature and mineralogical composition on bending strength and porosity of ceramic tile bodies. Appl. Clay Sci..

[8] Baccour H., Medhioub M., Jamoussi F., Mhiri T. (2008). Influence of firing temperature on the ceramic properties of Triassic clays from Tunisia. J. Mater. Process. Technol..

[9] Ducman V., Škapin A.S., Radeka M., Ranogajec J. (2011). Frost resistance of clay roofing tiles: Case study. Ceram. Int..

[10] Wiśniewska K., Pichór W., Kłosek-Wawrzyn E. (2021). Impact of the addition of dolomite to cream-firing clays on the technological and color properties of sintered ceramics. Int. J. Appl. Ceram. Technol..

[11] Kłosek-Wawrzyn E., Bugaj A. (2016). Influence of dolomite addition to masses made from triassic clay on application properties, phase composition and microstructure of ceramic materials produced. Ceram. Mater..

[12] Wyszomirski P. (2015). Ił z Borkowic (rejon opoczyński) jako wartościowy surowiec wielu dziedzin przemysłu ceramicznego (Borkowice clay (Opoczno region) as a valuable raw material in many areas of the ceramics industry). Zesz. Nauk. Inst. Gospod. Surowcami Miner. Energią Pol. Akad. Nauk.

[13] Weisser P., Lech R., Grabski J. (2014). Badanie właściwości dolomitów z trzech złóż, przeznaczonych do przemysłowego stosowania (The examination of the proprieties of dolomites from three deposits, used in the different industries). Cem. Wapno Beton.

[14] Zarubica A., Miljkovic M., Purenovic M., Tomic V. (2005). Colour parameters, whiteness indices and physical features of marking paints for horizontal signalization. Facta Univ.-Ser. Phys. Chem. Technol..

[15] Hiller S. (2000). Accurate quantitative analysis of clay and other minerals in sandstones by XRD: Comparison of a Rietveld and a reference intensity ratio (RIR) method and the importance of sample preparation. Clay Miner..

[16] Stoch L. (1988). Metody Badań Minerałów i Skał: Metody Termiczne.

[17] Lech R. (2008). Termiczny Rozkład Wapieni: Transport Masy i Ciepła.

[18] Salvator A.R., Calvo E.G., Aparicio C.B. (1989). Effects of sample weight, particle size, purge gas and crystalline structure on the observed kinetic parameters of calcium carbonate decomposition. Thermochim. Acta.

[19] Abubakar M., Muthuraja A., Ahmad N. (2021). Experimental investigation of the effect of temperature on the density of kaolin clay. Meter. Today Proc..

[20] Niesyt M., Psiuk B. (2017). Fused dolomite-magnesia co-clinker for fired dolomite refractories. Ceram. Int..

[21] Wang W., Shin Z., Wang X., Fan W. (2016). The phase transformation and thermal expansion properties of cordierite ceramics prepared using drift sands to replace pure quartz. Ceram. Int..

[22] Redaoui D., Sahnoune F., Heraiz M., Saheb N. (2018). Phase formation and crystallization kinetics in cordierite ceramics prepared from kaolinite and magnesia. Ceram. Int..

[23] Njoya D., Elimbi A., Fouejio D., Hajjaji M. (2016). Effects of two mixtures of kaolin-talc-bauxite and firing temperatures on the characteristics of cordierite- based ceramics. J. Build. Eng..

[24] Sembiring S., Simanjuntak W., Situmeang R., Riyanto A., Sebayang K. (2016). Preparation of refractory cordierite using amorphous rice husk silica for thermal insulation purposes. Ceram. Int..

